# P13 Systematic review assessing the impact of using McIsaac and Centor scores to aid antibiotic prescription decision making in patients presenting to secondary care with pharyngitis

**DOI:** 10.1093/jacamr/dlae136.017

**Published:** 2024-08-23

**Authors:** Atchchuthan Kanagasabai, Callum Evans, Hayley E Jones, Alastair D Hay, Sarah Dawson, Jelena Savović, Martha M C Elwenspoek

**Affiliations:** Population Health Sciences, Bristol Medical School, University of Bristol, Bristol BS8 2PS, UK; Population Health Sciences, Bristol Medical School, University of Bristol, Bristol BS8 2PS, UK; Population Health Sciences, Bristol Medical School, University of Bristol, Bristol BS8 2PS, UK; Centre for Academic Primary Care, Bristol Medical School, University of Bristol, Bristol, UK; Population Health Sciences, Bristol Medical School, University of Bristol, Bristol BS8 2PS, UK; The National Institute for Health Research Applied Research Collaboration West (NIHR ARC West), University Hospitals Bristol and Weston NHS Foundation Trust, Bristol BS1 2NT, UK; Population Health Sciences, Bristol Medical School, University of Bristol, Bristol BS8 2PS, UK; The National Institute for Health Research Applied Research Collaboration West (NIHR ARC West), University Hospitals Bristol and Weston NHS Foundation Trust, Bristol BS1 2NT, UK; Population Health Sciences, Bristol Medical School, University of Bristol, Bristol BS8 2PS, UK; The National Institute for Health Research Applied Research Collaboration West (NIHR ARC West), University Hospitals Bristol and Weston NHS Foundation Trust, Bristol BS1 2NT, UK

## Abstract

**Background:**

Complications can develop in the 5%–30% of pharyngitis cases caused by group A streptococcus (GAS); therefore, the appropriateness of antibiotics needs to be considered. Centor and McIsaac scores are clinical prediction rules for diagnosing GAS pharyngitis. Antibiotic prescription score thresholds vary between guidelines.

**Objectives:**

Estimate the sensitivity and specificity of McIsaac and Centor scores to diagnose GAS pharyngitis and evaluate their impact on antibiotic prescribing under different testing scenarios at each threshold in secondary care.

**Methods:**

MEDLINE, Embase and Web of Science were searched from inception to September 2022 for studies of patients presenting with acute pharyngitis to emergency or outpatient clinics that estimated the accuracy of McIsaac or Centor scores against throat cultures and/or rapid antigen detection tests (RADT) as reference standards. Quality Assessment of Diagnostic Accuracy Studies (QUADAS-2) was used for risk of bias assessment. Sensitivities and specificities of McIsaac and Centor scores were pooled at each threshold using bivariate random effects meta-analysis. We described antibiotic prescription rates under different testing scenarios and at different thresholds using natural frequencies for 10 000 patients.

**Results:**

Fourteen studies were included (eight McIsaac and six Centor scores). Eight studies had unclear and six had high risk of bias. McIsaac score had higher estimated sensitivity and lower specificity relative to Centor scores at equivalent thresholds but with wide and overlapping confidence regions. Using either score as a triage to RADT to decide antibiotic treatment would reduce antibiotic prescriptions relative to RADT test on everyone: estimated reductions in prescriptions in a cohort of 10 000 patients 1244, 172 and 666 for NICE (Centor), Danish and German (McIsaac) guidelines respectively, but with 893, 87 and 368 of these being true positive GAS cases.

**Conclusions:**

Centor and McIsaac scores are equally ineffective at triaging patients who need antibiotics. Finding a balanced treatment threshold to recommend prescribing antibiotic to prevent over/under treatment is challenging. Good safety netting by clinicians may enable use of high treatment thresholds safely and prevent over-prescription of antibiotics.

